# Reptiles on the wrong track? Moving beyond traditional estimators with dynamic Brownian Bridge Movement Models

**DOI:** 10.1186/s40462-020-00229-3

**Published:** 2020-10-27

**Authors:** Inês Silva, Matt Crane, Benjamin Michael Marshall, Colin Thomas Strine

**Affiliations:** 1 Conservation Ecology Program, School of Bioresources and Technology, King Mongkut’s University of Technology Thonburi, Bangkhunthien, Bangkok, Thailand; 2 School of Biology, Institute of Science, Suranaree University of Technology, Nakhon Ratchasima, Thailand

**Keywords:** Reptile, Simulation, Spatial ecology, Minimum convex polygon, Kernel density, Dynamic Brownian Bridge Movement Models, Snake, Lizard, Squamate

## Abstract

**Background:**

Animal movement expressed through home ranges or space-use can offer insights into spatial and habitat requirements. However, different classes of estimation methods are currently instinctively applied to answer home range, space-use or movement-based research questions regardless of their widely varying outputs, directly impacting conclusions. Recent technological advances in animal tracking (GPS and satellite tags), have enabled new methods to quantify animal space-use and movement pathways, but so far have primarily targeted mammal and avian species.

**Methods:**

Most reptile spatial ecology studies only make use of two older home range estimation methods: Minimum Convex Polygons (MCP) and Kernel Density Estimators (KDE), particularly with the Least Squares Cross Validation (LSCV) and reference (*h*_*ref*_) bandwidth selection algorithms. These methods are frequently applied to answer space-use and movement-based questions. Reptile movement patterns are unique (e.g.*,* low movement frequency, long stop-over periods), prompting investigation into whether newer movement-based methods –such as dynamic Brownian Bridge Movement Models (dBBMMs)– apply to Very High Frequency (VHF) radio-telemetry tracking data. We simulated movement data for three archetypical reptile species: a highly mobile active hunter, an ambush predator with long-distance moves and long-term sheltering periods, and an ambush predator with short-distance moves and short-term sheltering periods. We compared traditionally used estimators, MCP and KDE, with dBBMMs, across eight feasible VHF field sampling regimes for reptiles, varying from one data point every four daylight hours, to once per month.

**Results:**

Although originally designed for GPS tracking studies, dBBMMs outperformed MCPs and KDE *h*_*ref*_ across all tracking regimes in accurately revealing movement pathways, with only KDE LSCV performing comparably at some higher frequency sampling regimes. However, the LSCV algorithm failed to converge with these high-frequency regimes due to high site fidelity, and was unstable across sampling regimes, making its use problematic for species exhibiting long-term sheltering behaviours. We found that dBBMMs minimized the effect of individual variation, maintained low error rates balanced between omission (false negative) and commission (false positive), and performed comparatively well even under low frequency sampling regimes (e.g., once a month).

**Conclusions:**

We recommend dBBMMs as a valuable alternative to MCP and KDE methods for reptile VHF telemetry data, for research questions associated with space-use and movement behaviours within the study period: they work under contemporary tracking protocols and provide more stable estimates. We demonstrate for the first time that dBBMMs can be applied confidently to low-resolution tracking data, while improving comparisons across regimes, individuals, and species.

**Supplementary information:**

**Supplementary information** accompanies this paper at 10.1186/s40462-020-00229-3.

## Introduction

Animal movement is an underlying process in many ecological systems, and there is a growing understanding of how individuals behave through space and time [1, 2]. Movement is often conceptualized then presented as a home range, defined as the area animals move through during “normal” activities, including resource acquisition and reproduction [3, 4]. While the utility of the home range concept has been questioned in recent years [5, 6], its estimation continues to have a wide range of applications, such as identifying behavioural adaptations to predictable environmental features [7] or inferring habitat use [8–11]. It is clear that different biological questions have different appropriate estimators [12], but reptile spatial ecology studies evaluate not only long-term area requirements, but also movement behaviour and space-use regardless of the assumptions and applicability of each estimation method. In this paper, we focus on the concept on space-use and movement pathways during the sampling period, when “occurrence distribution” methods are appropriate. This definition is not synonymous with the stricter definition of home range, that seeks to predict future animal space-use beyond the sampling period (“range distribution”, i.e. the space required for an entire animal’s life-stage sensu [3]). Applying a space-use approach to ecological research questions requires careful consideration [13], as any conclusions drawn are profoundly impacted by the natural history of the target species.

Terrestrial reptiles —broadly lizards, snakes, and tortoises— have *distinct* natural histories from mammals (e.g., ectothermic thermoregulation demands, long periods of digestion, ecdysis), resulting in *distinct* movement patterns. Many reptiles move less frequently than comparatively sized mammals [14], but more importantly, many terrestrial reptiles spend prolonged periods stationary under shelter (1 day to several weeks, see [15–17]). These inconsistent movement patterns severely impact inferences drawn from estimation methods. Traditional home range estimators –Minimum Convex Polygons (MCP) and Kernel Density Estimators (KDE)– continue to be mainstream in recent literature but present major limitations for telemetry-based reptile studies: MCPs provide only an outline of an individual’s outer-most movements, including large areas never used by the animal and ignoring any selection patterns, while KDEs include parameter choices that severely affect overall area estimates and assume independence which is breached by movement data [18, 19]. With the rise of Global Positioning System (GPS) animal tracking, researchers have developed new statistical approaches for calculating potential space-use, taking advantage of the high number of location fixes and explicitly incorporating the serial autocorrelation of movement data.

Dynamic Brownian Bridge Movement Models (dBBMMs) are a technique intended for GPS telemetry, allowing for efficient and repeatable analysis of high-resolution data –particularly useful for animals with behaviourally distinct movement patterns. Although dBBMMs are not appropriate for home range estimations, they can be applied to specific research questions, if the data is sampled coarsely or for short study periods [12]. The method creates a one-dimensional fix-frequency independent behavioural measure (Brownian motion variance [20]) that has been employed to elucidate avian and mammal movement patterns and provides a confidence region of where the movement pathways fall (e.g., [21–24]). While tracking reptiles with GPS may currently be limited (see [25–28]) by their natural history [29, 30] —e.g. weakened signal due to the surgical implantation, attachment of the tag, limited number of species which can be ethically attached due to body size [28], reduced fix rates and precision due to underground sheltering [16, 30]— leveraging dBBMMs may still benefit reptile VHF studies [31, 32]. Multiple simulations studies have investigated how different methods interact with animal movement and space-use delineation (e.g., [18, 33–35]), but none have targeted reptile-specific movement patterns. In a previous paper [31], we provided proof of concept for choosing dBBMMs over MCPs and KDEs for a single study species (*Ophiophagus hannah*) with VHF data; note, in [31] we incorrectly refer to dBBMMs as a home range method. Subsequently, other studies have adopted dBBMMs to assess reptile spatial ecology with VHF data (4 snake species, 1 tortoise); however, widespread application of dBBMMs remains limited as all studies originated from a single site in Northeast Thailand. While these studies have shown that dBBMMs can perform well with reptile VHF tracking data, the wider application of the technique requires further exploration. We must tailor our methodologies to the peculiarities of reptile movement, and assess the utility of newer methods to properly inform desperately needed conservation actions [36, 37].

As such, we assess estimation methods of space-use and movement pathways (specifically those targeting within-sample interpolation), resulting from variable study designs common in the reptile spatial ecology literature: namely temporally low-resolution tracking regimes. We simulate movement data of three archetypal reptile species, thoroughly examining two common” range distribution” estimators, MCPs and KDEs. Next, we compare these traditional estimators to a newer “occurrence distribution” method: dBBMMs. Finally, we discuss the implications of estimator choice regarding space-use and movement pathways, and present guiding principles for reptile spatial ecology sampling designs that wish to address these research questions.

## Materials and methods

### Simulated animal movement and tracking data

We used the *SimData* function in the *momentuHMM* package [38] to simulate movement data from a Hidden Markov Model (HMM). HMMs are time-series models where the movement pattern of an animal is assumed to depend on the underlying behavioural state of the animal [39]. We simulated data for 32 individuals from three archetype reptile movement patterns, to represent three main groups within reptile movement ecology (for simplicity, we refer to archetypes as species):

**Species 1** corresponds to highly mobile (active hunters) with long-term shelter sites (e.g., monitor lizards, some skinks, and elapids like mambas and king cobras). Highly mobile reptile species are usually active hunters (i.e., forage by actively searching for prey), as they show higher values of standard metabolic rates than ambush predators [40]. Foraging activity can vary widely among active hunting snake species, depending on the kind of prey hunted [40], we chose to reflect a specialization on larger prey as it may be favoured over small prey items (e.g., [41, 42]). As such, this will lead to longer use of sheltering sites (for thermoregulation and digestion of prey items).

**Species 2** represents less mobile reptiles, capable of moving long distances but are ambush foragers, and will still shelter for long periods (e.g., pythons). Sedentary, “sit-and-wait” ambush predators include many snake species, such as vipers, pythons, boas, as well as some colubrids and elapids [40, 43]. Snakes with an ambush foraging strategy consume a wide range of meal sizes [44]: We chose to model Species 2 after a specialization on larger prey and thus longer digestion periods. The main component of an ambush predator is spending long periods in the same location [45, 46]. “Sit-and-wait” predators also usually feed less frequently than active hunters [47].

**Species 3** represents smaller ambush predators, infrequently moving and sheltering for shorter periods (e.g., vipers, some smaller lizard species). In contrast to Species 2, we chose to model Species 3 with a smaller body mass, reflected by short-distance moves and specialization on smaller prey (resulting in shorter sheltering events, as digestion is faster with smaller prey items). In particular, Species 3 behaves similarly to certain pit vipers (family Viperidae): ambush predators, with low energetic requirements, infrequent feeding events, as well as a reduced movement rate and home range size compared to larger snake species [48, 49].

We have attempted to capture the diversity of reptile movements, but not all species will fit within these archetypes; many species will occur between these types, and some cases exist where movement closely matches mammalian patterns (i.e., limited long stationary periods, with periodic daily movement and foraging bouts). Each archetype had a unique set of state-dependent parameters and transition probabilities with the same three behaviour states: “sheltering” (state 1), “moving” (state 2), “resting” (state 3). “Sheltering” behaviour includes long stationary periods, reflecting periods of prolonged digestion. “Moving” includes discrete periods of movement (species-dependent step lengths), indicative of searching behaviour for prey, mates, and shelter sites. “Resting” covers regular, but not prolonged, stationary periods driven by the circadian rhythm; resting behaviour also allows for smaller nocturnal movements distinguishing it further from sheltering. The state-dependent data streams included step length (l_*t*_) and turning angle (θ_*t*_), which we generated from Gamma and von Mises distributions, respectively. We selected Gamma parameters to strictly reflect the monotonically decreasing density function for state 1 and 3 (“sheltering” and “resting”), and a density function with mode distinct from zero for state 2 (“moving”). Then we adjusted Gamma (μ, σ) for state 2 between each archetype and set it on a relative scale to the simulated landscape to reflect their distinct movement patterns (e.g., long versus short moves). The von Mises distribution has its mean centered around zero (α ~ 0) but with very weak concentration (κ ~ 0.01) to reduce any strong persistence in movement direction. The simulations included a spatially correlated covariate for state 2, to reflect habitat preferences, while states 1 and 3 followed a *cosinor* function, to reflect cyclical patterns of long-term sheltering (state 1) and circadian rhythms (state 3). To simulate individual variation and movement in a heterogeneous landscape, we generated a random neutral landscape with fractal Brownian movement, using the *NLMR* package [50]. For further details on these simulated species, as well as their specific step lengths, turning angles and transitional probabilities, see Additional File 1.1.

**Fig. 1:**
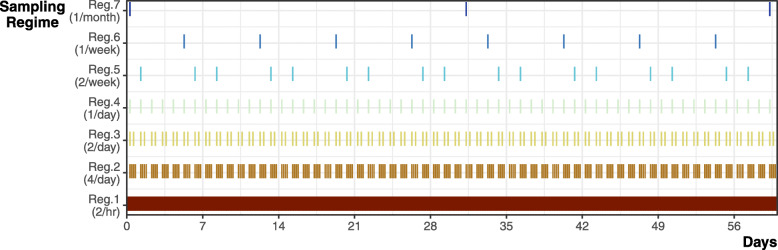
Example two-month period showing how data is thinned to represent different tracking regimes

After creating the full simulated data set (regime 1), we generated six subsets of the data to represent various field sampling regimes (regime 2–7): four locations per day, two locations per day, one location per day, two locations per week, one location per week, and one location per month (Fig. 1). For each subset, we assumed a consistent regularly scheduled sampling protocol limited to the species’ activity periods.

The autocorrelated nature of tracking data poses difficulties for estimator methods that assume independence between points, namely KDEs. Attempting to remove autocorrelation to fit these assumptions can reduce the biological relevance of the space-use estimates [51], but it is still advocated in reptile spatial ecology studies [52, 53]. We investigated the temporal autocorrelation present in our simulated dataset to determine whether our coarser sampling regimes complied with KDE independence assumptions. Other than less frequent tracking, autocorrelation may be reduced by removing repeated locations, a method of particular relevance for reptiles that exhibit long term sheltering. We considered this special case –sampling regime 8– where only animal relocations are included in the area estimation, by using the four location per day sampling regime as its starting point and then removing data points where the animal was stationary. We described the autocorrelation in the simulated data using the *ctmm* package’s variogram functionality [54], and plotted the minimum number of days until the autocorrelation became insignificant with raincloud plot code from Allen et al. [55].

### Estimator methods

#### Minimum convex polygon

We calculated the Minimum Convex Polygon (MCP) for each simulated individual that created the smallest area convex polygon containing all animal locations. We used the 95% MCP, which removes outlying points on the assumption that these represent exploratory movements and thus not part of the home range (as originally defined by [3]) or, in our case, representative of within-sample space-use. The MCP method has long been lauded as a way of maintaining comparability and historical consistency with previous studies [56], yet has well documented issues: extreme sensitivity to sampling size and tracking duration [57], and overestimated boundary delineation [58], with the inclusion of areas that the animals never use [59, 60]. However, Row and Blouin-Demers [18] argued that MCPs are preferable to kernel density estimators specifically for herpetofauna, and MCPs’ use persists for comparisons in reptile telemetry studies [61]. An additional and considerable limitation of MCPs is that they do not create a probabilistic utilization distribution.

#### Fixed kernel home range

Fixed KDE home ranges rely on a smoothing parameter (bandwidth, *h*) to generate a utilization distribution. Bandwidth selection for KDE can dramatically influence area estimation [62], and thus we included two bandwidth selection algorithms, reference bandwidth (*h*_*ref*_) and Least-Squares Cross-Validation (LSCV), for our comparisons. Both bandwidth selection methods are frequently used in reptile VHF studies, but potentially flawed for herpetofauna [18]. The *h*_*ref*_ method tends to overestimate areas while LSCV tends to underestimate [63]. In general, fixed KDE home ranges are not accurate when using autocorrelated data regardless of bandwidth selection function [64].

#### Dynamic Brownian Bridge Movement Model

Dynamic Brownian Bridge Movement Models (dBBMMs) provide occurrence distributions based on animal movement paths [20]; from these occurrence distribution we can draw confidence regions as estimates of potential animal movement pathways and space-use during the study period. The method accounts for temporal autocorrelation, so it requires all locations to be time stamped. In addition, dBBMMs incorporate error associated with each triangulated location, which we kept consistent across species and regimes (at 5 m) for the following reasons: (1) neither MPCs nor KDEs account for location error, so the evaluation of the impact of this metric would be solely on one method and not effective for comparison purposes; (2) location error associated with VHF telemetry is extremely variable, dependent on macro and micro-habitat characteristics as well as tracking protocols (which we are not assessing); and (3) we wanted to account for cases where GPS error can be greater than step length (e.g., vipers, small lizards). The dBBMM method also allows calculation of Brownian motion variance (*σ*^*2*^*m*), which can help researchers determine how movement trajectories can occur due to a species’ behaviour and activity [20]. Motion variance can help detect breeding and foraging behaviour in reptiles, even with VHF telemetry data [31].

### Method comparison

To compare the error generated from each estimator, we calculated the overlap with the theoretical “true movement pathway” for each individual (i.e., the true path the animal used during the study period). We generated an individual’s “true movement pathway” by creating a buffer around all the simulated movement points with a width of two-times the step length intersect from each simulated species’ movement state (40-m for Species 1, 20-m for Species 2, 10-m for Species 3). This provided a conservative confidence region (excluding the impact of habitat) around the trajectory, but more generous and biologically sensible than only using simulated straight line movement pathways. For each area estimation, we calculated the commission (Type I, false positive) and omission (Type II error, false negative), using the 95% contours for MCP, KDE and dBBMMs. We used the 95% contours, as this is the standard level used in most spatial ecology studies, and prevents estimations from being overly impacted by outlying movements. We then calculated the F-measure [2/(recall^− 1^ + precision^− 1^)], where precision (also known as the positive predictive value) is the area of true positives over the area of true negatives and positives, and recall (also known as sensitivity) is the area of true positives over the area of true positives and false negatives. F-measures provide a balanced metric of false positive and false negative, while being insensitive to true negative rates [65]. The values of F-measures reflect model accuracy; a low F-measure indicates low model accuracy, and an F-measure of 1 indicates excellent precision and recall.

We explored the relationship between methods, regimes, and F-measures using a Bayesian generalized linear mixed model with the *brms* package [66]. We specified a model set for each species, with F-measure as our response variable following a beta distribution (as it is bound between 0 and 1), with individual as a random effect to account for individual variation, and a varying slope for the effect of method. We excluded regime 8 (four locations a day, relocations only) as this sampling regime was not systematic. We ran models with six Markov Chain Monte Carlo (MCMC) chains, each with 6000 iterations (1000 burn-in iterations, thin = 1), and we set Δ to 0.99. We fitted each model with half-Cauchy weakly informative priors [67]. We checked model convergence by inspecting trace plots and $\hat{R}$ values [66], assessed model fit visually via posterior predictive diagnostic plots, and evaluated model performance using leave-one-out cross-validation [57] and Bayesian *R*^*2*^. For further details on model selection and validation, see Additional File 1.2.

We compared the special case of regime 8 (like regime 2 but only relocation points) to the original regime 2 in its own Bayesian model set; this allowed us to evaluate the impact of removing stationary locations as a method of reducing data autocorrelation. Additionally, for this special case we only compared the best performing KDE bandwidth (LSCV) and dBBMMs.

We wrote code for R v.3.5.2 [68] using R studio v.1.2.1335 [69], and made all data and code available at 10.5281/zenodo.3660795.

## Results

### Simulated animal movement and tracking data

**Fig. 2:**
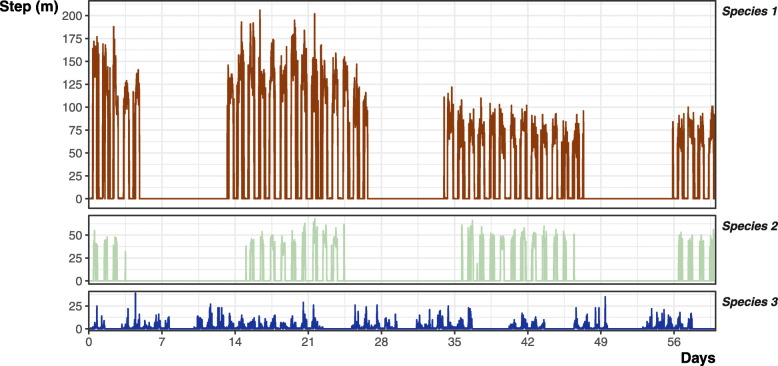
Example two-month period illustrating how step distance (m) and its frequency differs between our three species archetypes

The complete dataset for each simulated individual consisted of *n* = 17,521 data points for a full year, with 30-min time steps (regime 1). Each regime progressively lowered the available data (*n*^reg 2^ = 1460 data points, *n*^reg 3^ = 730, *n*^reg 4^ = 365, *n*^reg 5^ = 104, *n*^reg 6^ = 52, *n*^reg 7^ = 12), while regime 8 varied for each species and individual due to the variability in sheltering and resting behaviour (*n*^species 1^ *=* 5189 ± 204 data points (mean ± SD); *n*^species 2^ *=* 3501 ± 1099; *n*^species 3^ *=* 3873 ± 573). Visual validation of movement patterns matched with reported patterns in the literature (e.g., [11, 28, 31, 70–74]), and the predicted patterns of the three archetypes (Fig. 2).

**Fig. 3:**
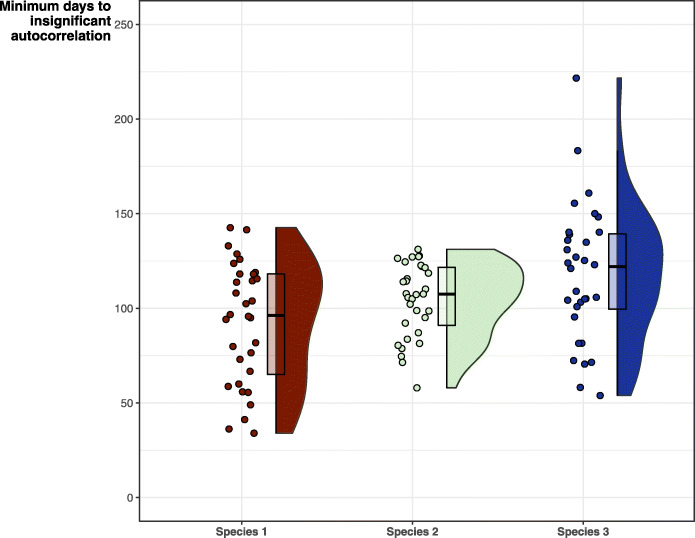
Minimum number of sampling days until the autocorrelation becomes insignificant and data points can be considered independent

As expected, all simulated species and individual datasets showed strong autocorrelated structure. Time until insignificant autocorrelation far exceeded even the coarsest tracking regime tested (regime 7, i.e.*,* 1/month), indicating that all tracking regimes breach the assumption of independence required for KDE methods (Fig. 3).

### Method comparison: omission vs. commission

**Fig. 4:**
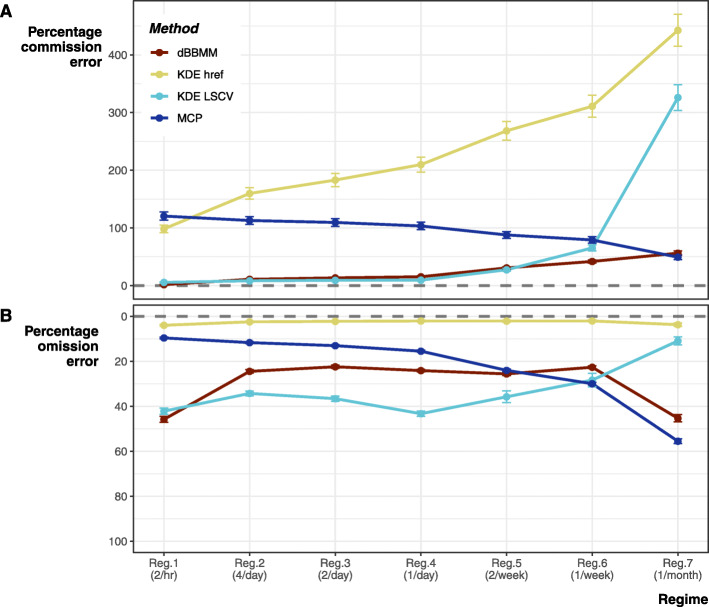
Percentage error from the true movement pathway using 95% contours. **a** Commission error (Type I, false positive), and **b** omission error (Type II, false negative). Error bars represent standard error (SE) of means across species (3) and individuals (96). Note, panels **a** and **b** have different scales for error because omission error cannot exceed 100% of the true movement pathway

Overall, coarser tracking regimes lead to greater % error when compared to the true movement pathway. However, the balance between commission (Type I, false positive) and omission (Type II, false negative) is inconsistent and varies between estimation methods (Fig. 4). There is also a general trend towards commission error when estimating areas because omission error is bounded between 0 and 100%.

#### Minimum convex polygon

**Fig. 5:**
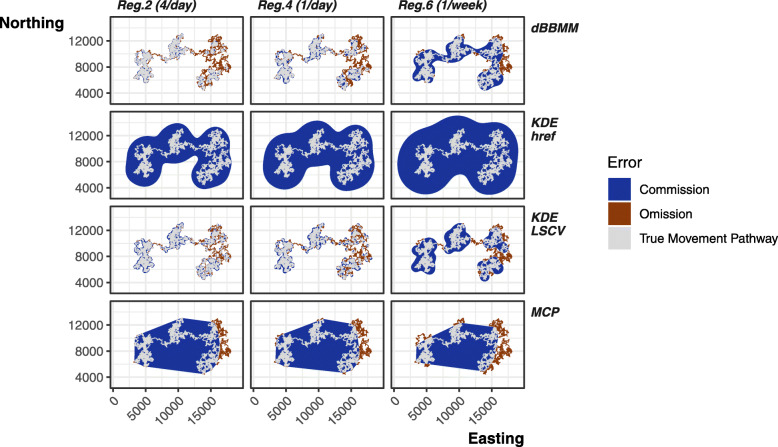
An example individual from species 1 showing how method and regime can interact to produce different levels of commission (Type I, false negative; blue areas), and omission (Type II, false positive; red areas) error compared to the true movement pathway (grey areas). All contours shown are produced from the 95% contours. Example individuals from species 2 and 3 are in Additional file 1.3

Minimum convex polygons were the only method that showed a constant offset between omission and commission, as one increases the other decreases nearly 1:1. In addition, MCPs were the only method that decreased their commission error as tracking regime became temporally coarser. At frequent tracking regimes, MCPs only introduced minimal omission error, but their starkest failure is in their simple shape leading to the greatest commission error at highest resolution tracking regime (Figs. 4, and 5).

#### Fixed kernel home range

The fixed kernel density estimation using *h*_*ref*_ smoothing factor was by far the worst estimator for commission error. At low resolution tracking regimes, the > 400% overestimation leads to near complete loss of area edge fidelity (Fig. 4). Due to this heavy emphasis on generous area estimation KDE *h*_*ref*_ produced negligible omission error.

By comparison KDE LSCV produced consistently lower commission error at higher resolution tracking regimes, but once the regime was once a week or coarser LSCV commission error spikes (up to 300% overestimation). LSCV consistently performed worse in terms of omission error when applied to tracking regimes with multiple tracks per day. Additionally, the LSCV algorithm frequently failed to converge, i.e., could not determine the optimal smoothing value (68.5% of all LSCV home ranges failed). Only regime 7 converged consistently; the inclusion of more data exacerbated convergence failure (regime 1–4, 100%; regime 5, 43.8%; regime 6, 33.3%). Using only relocations reduced convergence failures (regime 8, 2.08%) compared to its closest parallel regime (regime 2, 100%). For both KDE methods, omission and commission error variability (displayed as SE on Fig. 4) increased as tracking regime became coarser.

#### Dynamic Brownian Bridge Movement Model

Overall dBBMMs performed best by accurately representing the true movement pathways. The method produced low commission error levels, matching KDE LSCV performance (Fig. 4). Unlike LSCV, dBBMMs commission error remained more stable and lower when applied to coarser tracking regimes. Only MCPs produced a comparative level of commission error at the coarsest tracking regimes, but dBBMMs kept some semblance of shape fidelity and connectivity (Fig. 5). Unlike other methods, dBBMM error remained low and balanced between omission and commission, never exceeding 75%.

#### Special case of regime 8

**Fig. 6:**
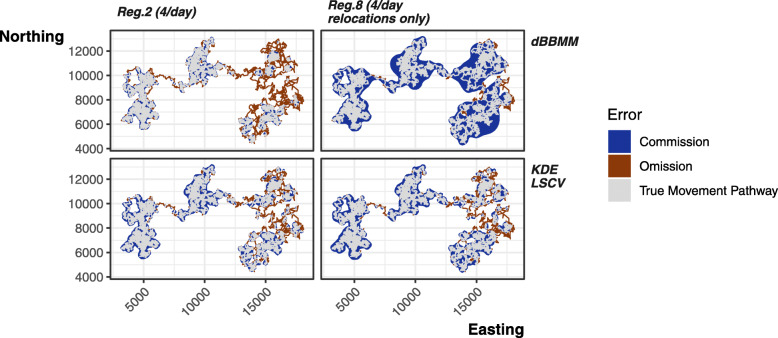
Comparison between the commission (Type I, false positive; blue areas) and omission (Type II, false negative; red areas) error rates produced by the KDE LSCV and dBBMM 95% contours when using data from sampling regime 2 (four locations per day) and regime 8 (four locations per day, relocations only)

Tracking regime 8 cannot be directly compared to the other regimes as the structure of the tracking is different. A fairer comparison is between regime 8 (four locations per day, relocations only) and regime 2 (four locations per day). Like all other regimes, regime 8 fails to remove autocorrelation to insignificance (Fig. 3); however, it did improve the performance of KDE LSCV estimation despite still breaching the fundamental independence assumption (Figs. 5, 6). The removal of repeated stationary points prevented the LSCV smoothing from grouping too tightly to point concentrations (i.e., long-term shelter sites), ultimately countering the tendency towards omission error for LSCV. On average, dBBMMs performed very similarly and balanced the omission and commission well (Fig. 4). The dBBMMs had the added advantage of assuming serial dependence of points and, unlike LSCV, performed well when provided low or high quantities of data.

### Method comparison: F-measures

**Fig. 7:**
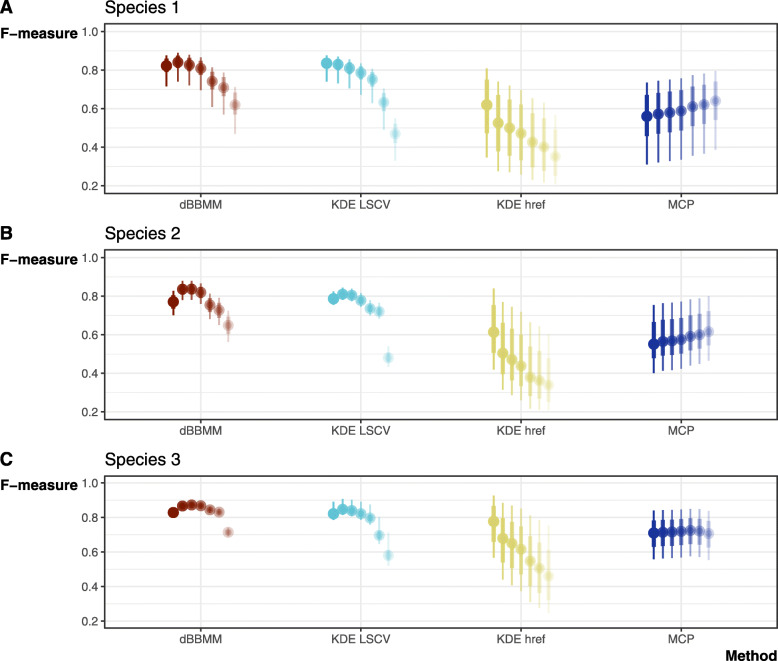
Model results that aimed to predict F-measures using method, regime, and individual ID by species. Tracking regime 1–7 are shown left to right with lowering levels of opacity. Fitted draws were taken only from the first 5000 samples

The Bayesian models converged and performed well for all three species, with $\hat{R}$ values ≃ 1.00 (Additional File 1.2), and *R*^*2*^ values indicating considerable predictive power (Species 1: Bayesian *R*^*2*^ = 0.960, 95% CrI: 0.958–0.962; Species 2: Bayesian *R*^*2*^ = 0.946, CrI: 0.755–0.786; Species 3: Bayesian *R*^*2*^ = 0.905, CrI: 0.897–0.911). Overall, our best models showed an interaction effect of methods and regimes on F-measures; all species had a non-zero positive relationship between F-measures and regimes, with higher estimates for dBBMM and KDE LSCV, while both MCP and KDE *h*_*ref*_ showed considerably worse F-measures (Fig. 7). However, Species 1 area estimations were associated with lower F-measures, suggesting that the potential space-use and movement pathways of species with high movement and long periods of sheltering are harder to model than those with more stable movement patterns.

## Discussion

Both MCPs and KDEs produced high error rates and failed to properly reflect simulated reptile movement pathways, which is particularly concerning because of their widespread use. While originally intended for GPS telemetry, we found that dBBMMs performed well across a range of lower fix rates sampling regimes, and for our three archetypical reptile species. In fact, although Brownian Bridge estimators become singularly defined upon the observed movement pathway for high-frequency GPS data [12], they provide a fairly accurate and stable confidence region for coarse, low-frequency VHF datasets. Particularly for species without range residency, or with short duration studies, where newer methods such as the autocorrelated kernel cannot be applied [12, 75], or when the chosen research questions focus only on within-sample interpolation. Both KDE smoothing parameter selection techniques produced high error rates with lower frequency fix rates when compared with dBBMMs, casting doubt on the ability of KDEs to provide comparable estimates between studies focusing on within-sample space-use or movement pathways; particularly if these studies employ different tracking protocols.

Our low-resolution tracking regimes generated a varied number of data points, ranging from only 12 to at most 1460 fixes, representing 0.07–8.30% of the initial full data set of locations (i.e., regime 1; a location every 30 min). The number of fixes can dramatically impact area estimates, and thus Seaman et al. [76] suggested a minimum of 30–50 locations before applying KDEs, while Girard et al. [77] suggested an even more stringent criteria, recommending at least 300 locations. Whereas regime 7 (one location per month; *n* = 12) failed the first criteria, regimes 5–7 (at least two locations per week or coarser; *n* ≤ 104) all failed to meet the stricter requirements, indicating that common reptile tracking regimes fail to meet the initial assumptions of KDEs. Removing non-relocations (regime 8) also reduced fixes by 70.4–80.0%, worsening area estimates, while ultimately failing to properly address the additional assumption of independence required for KDE methods. Furthermore, this fix diverts attention from what should be the ultimate goal of any estimation method—i.e., obtaining a representative sample of locations from a targeted individual in accordance to the chosen research question— instead of simply ensuring data independence [19], for either within-sample interpolation or beyond-sample extrapolation.

The use of MCP and KDE *h*_*ref*_ produced large commission (false positive) errors, which if carried forward are liable to impact movement behaviour and space-use inferences [19, 78]. By comparison, both KDE LSCV and dBBMM estimations fared better, although LSCV failed to produce F-measures comparable to dBBMMs under low-resolution tracking regimes. As a fix-frequency independent method [20], dBBMMs performed most consistently across sampling regimes with the lowest error rates, even in low-resolution datasets, improving on both traditional MCP and fixed KDE methods. To match dBBMM performance at the sparsest regimes (*n* = 12) KDEs required four times the data. Maximizing performance under low-resolution regimes is critical for VHF studies because the data are time, effort, and cost intensive [79]. Furthermore, dBBMMs require no a priori knowledge of an animal’s movements (necessary to identify the correct smoothing bandwidth for KDEs), and can be put to use with current telemetry practices or to re-analyse previously collected data. The dBBMM method is easily compatible with low-resolution data from herpetofauna spatial ecology studies still reliant on VHF, representing a cheap and immediate alternative to long-term high-resolution tracking methods (GPS) that remain elusive for herpetofauna [30, 80]. Presently, applications of dBBMMs to reptile movement data are still restricted to a single field site [31, 74].

Although KDE LSCV came closest to performing comparably with dBBMMs at high resolutions, beyond failing the initial point independence assumption, this smoothing parameter failed to converge. In other words, the LSCV method could not identify the optimal smoothing factor, making the estimations unstable and unusable (supporting findings from [63]). Non-convergence issues are compounded by large numbers of identical locations or very tight clusters (i.e., high site fidelity), which we did not explicitly simulate. While ignoring side fidelity can inflate LSCV performance [62, 63, 76, 81], we demonstrate that dBBMMs still performed similarly or better than LSCV even in these suboptimal conditions. Only regime 8 (with removed non-relocations) improved KDE LSCV while hindering dBBMMs. However, as we previously mentioned, this fix compromises the biological relevance of area estimates as the autocorrelated nature of animal movement is inherently biologically relevant and we should not seek to eliminate the structure of our data [19, 51]. Explorations using real GPS data also show consistent problems with KDE LSCV omission error, leading to severe undersmoothing, and frequent convergence failures [63]. In addition, Jones, Marron, & Sheather [82] found that LSCV-smoothed utilization distributions had unacceptable variability, that can further undermine comparisons between individuals, populations, or studies.

Archetypal species movement characteristics influenced our estimates (MCP, KDE and dBBMM). The active hunter (Species 1), with its sporadic long-distance moves, had lower F-measures and higher error rates than the ambush predators (Species 2 and 3). Essentially, greater movement capability led to greater uncertainty concerning movement pathways (i.e., larger confidence region estimates from dBBMMs). When comparisons between species are required, researchers should explore how regime and estimation method affect comparisons. Ideally, researchers should be able to access the original data from previous studies to confidently compare between species. We encourage greater use of open data repositories in reptile studies (e.g., Movebank). Compared to other taxonomic groups, terrestrial reptile data on Movebank is currently lacking (7 species, 3 testudines and 4 snakes), out of over 950 species present on Movebank).

Reptile movement is unique compared to other taxonomic groups, as long-term sheltering essentially leads to a highly zero-inflated movement dataset, which introduces error in area estimates by under- and over smoothing with traditional estimators. While dBBMMs provide a more direct modelling approach for movements –a critical component of assessing habitat use [83]– they are limited to within-sample interpolation (i.e., not home range as defined by [3]). Other methods that tackle space-use in the sense of home range, such as the autocorrelated KDE [12], are more appropriate if the research question calls for the estimation of long-term area requirements or beyond-sample extrapolation. Within-sample space use and movement estimates can also be further enhanced by methods incorporating landscape (e.g., dBBMM with covariates [84]) or behaviour [85].

## Conclusions

Ultimately, accuracy of area estimates will be dependent on resources, tracking frequency and study duration [86], all directly impacting the viability of proposed telemetry studies. A clearly defined question [87] enables researchers to identify potential trade-offs in the right context, and should be considered during study design with specific consideration of the area of interest (within-sample space-use and movement pathways versus home range) and the appropriate estimation method. There will always be spatial uncertainty, but it should be minimized with reference to the chosen research question and any targeted behaviours [88, 89]. Better estimators are an inexpensive way to optimize returns from tracking data compared to technological advances or increasing fieldwork.

Our work concurs with previous studies e.g., [90] further reiterating known problems with both MCP and KDEs despite claims of continued use to maintain “comparability”. We find this deeply flawed, particularly in cases where tracking regimes or estimators differ, as they can produce dramatically different error rates. Instead, we demonstrate dBBMMs are more stable and accurate representations of movement pathways and thus suitable for proper comparisons. However, as an occurrence distribution method, dBBMMs should be applied only with the appropriate research questions (e.g., evaluating movement behaviour, pathways, and space-use within the study period). The information provided here can help optimise reptile spatial ecology by yielding more accurate and reproducible estimations.

## Supplementary information

**Additional file 1.**

## Data Availability

The datasets supporting the conclusions of this article and the R scripts used for all analyses are available in the Zenodo repository [10.5281/zenodo.3660795].
