# Supplementary material for: Reptiles on the wrong track? Moving beyond traditional estimators with dynamic Brownian Bridge Movement Models
Source: Mov Ecol. 2020 Oct 27;8:43. doi: 10.1186/s40462-020-00229-3 (PMC7592577; doi:10.1186/s40462-020-00229-3)
Supplement: Supplementary file 1 — Additional file 1. [file 40462_2020_229_MOESM1_ESM.docx]

# Reptiles on the wrong track? Moving beyond traditional estimators with dynamic Brownian Bridge Movement Models

Inês Silva, Matt Crane, Benjamin Michael Marshall, Colin Thomas Strine

# Additional file 1.1

Appendix S1 describes simulation inputs for the hidden Markov models (HMMs). We have defined three archetype species to represent three different groups within reptile movement ecology: **Species 1** corresponds to highly mobile reptiles (active hunters) with long-term shelter sites (*e.g.,* monitor lizards, some elapid snakes); **Species 2** represents less mobile reptiles, able to move long distances but with an ambush predator foraging strategy, which still require long-term shelter sites (*e.g.,* pythons, crocodiles); finally, **Species 3** represents smaller reptiles species, also ambush predators, with low likelihood of movement and shorter resting periods (*e.g.,* vipers, smaller lizards).

All three archetypes have different state-dependent parameters and transition probabilities, but exhibit the same three behaviour states: “sheltering” (state 1), “moving” (state 2), “resting” (state 3). “Sheltering” behaviour corresponds to relatively long periods of immobility, *i.e.,* periods of stasis after predation events, ambush or for thermoregulation (King & Duvall 1990, Webb & Shine 1997); “Moving” behaviour corresponds to discrete episodes of movement (species-dependent step lengths) when searching for prey, mates, ambush or shelter sites and retreats (King & Duvall 1990, Madsen & Shine 1993, Secor & Nagy 1994, Webb & Shine 1998) all reflecting, to some degree, habitat selection; “Resting” behaviour occurs only at night and corresponds to short periods of immobility associated with the circadian cycle (but still allowing for some potential shorter movement episodes throughout the night, unlike sheltering). We have created all our archetype species to be diurnal. The emphasis is not on the behaviour itself, but on modelling for space-use and movement pathways during the study period. The state-dependent data streams included step length ($l_{t}$) and turning angle ($ϴ_{t}$), which were generated from Gamma) and von Mises (α, κ) distributions, respectively. The state-dependent data streams included step length ($l_{t}$) and turning angle ($ϴ_{t}$), which were generated from Gamma and von Mises distributions, respectively:

| $l_{t} \vert S_{t}= s \sim gamma(\mu_{s},\sigma_{s})$, $\mu>0, \sigma>0$ | (1) |
| --- | --- |
| $ϴ_{t} \vert S_{t}= s \sim von Mises\left( \alpha_{s},\kappa_{s} \right), \alpha\epsilon\left( \pi, -\pi\right), \kappa>0$ | (2) |

We have modelled step length for state 2, “Moving” ($\mu_{t}^{l}$), as functions of the habitat value of each cell ($h_{t}$). This allows the simulated animal to showcase a pseudo-preference behavior, moving faster through bad habitats (*i.e.,* lower $h_{t}$) and slower through better habitats (*i.e.,* higher $h_{t}$):

| $\mu_{t}^{l}=\exp(\beta_{0}^{l}+\beta_{1}^{l}\times h_{t})$ | (3) |
| --- | --- |

The von Mises distribution has its mean centered around zero ($\alpha\sim0$), so there is persistence in the movement direction.

The transitional probability matrices, $\Gamma^{(t)}$, for all species follow the same format where each element$\gamma_{i,j}$ is the probability of switching from state *i* at time *t* to state *j* at time *t*+1 for *i*,*j* ∈ {1=“sheltering”, 2=“moving”, 3=“resting”'}:

| $\Gamma^{(t)}=\left( \gamma_{i,j}^{(t)} \right)=\left( \begin{matrix} \gamma_{1,2} & \cdots& \gamma_{3,j} \\ \vdots& \ddots& \vdots\\ \gamma_{1,2} & \cdots& \gamma_{3,j} \end{matrix} \right)$ | (4) |
| --- | --- |

Individuals cannot alternate between states 1 (“sheltering”) and 3 (“resting”) as we believe it is not biologically reasonable to go into sheltering or resting without first going through state 2 (“moving”), *i.e.,* without first exhibiting some foraging behaviour. This is reflected by a large negative number (-100), following recommendations from McClintock & Michelot (2018). We also modelled the transition probabilities to vary through time, with the transition of 1→2 and 2→1 being modelled as a *cosinor* function varying monthly (with slight changes by species). The transition of 2→3 and 3→2 we modelled as a *cosinor* function every 24 hours (daily; equal in all three species). This allows us to simulate patterns of both short-term resting (circadian rhythm; $\gamma_{1,2}$) and variable-length sheltering ($\gamma_{2,3}$). For the latter, $\tau_{i}$ is species-dependent to reflect different sheltering patterns:

| $\gamma_{1,2}^{(t)}=\gamma_{2,1}^{(t)}=[ cos (2\pi\times t/\tau_{i}), \sin(2\pi\times t/\tau_{i}) ]$, $t\in\left( 0,1,\ldots, 23 \right), \tau_{i}=24$ | (5) |
| --- | --- |
| $\gamma_{2,3}^{(t)}=\gamma_{3,2}^{(t)}=[ cos (2\pi\times t/\tau_{i}), \sin(2\pi\times t/\tau_{i}) ]$, $t\in(0,1,\ldots, 365)$ | (6) |

The low number of reptile studies using GPS technology, and inconsistent data accessibility, limited our ability produce literature-derived *a priori* estimations of step length and turning angles (see **Figure 2** in the main text, for step length over time for our three species). Therefore, we relied on the results of exploratory data analyses performed on simulations for each of our archetype species. By testing multiple parameters, we were able to identify those parameters that produced datasets approximating reality: Reality as inferred by the summary statistics provided in reptile telemetry studies.

## Species 1

## “*Active hunter, long-distance moves, long-term sheltering*”

Highly mobile reptile species are usually active hunters (*i.e.,* forage by actively searching for prey), as they show higher values of standard metabolic rates than ambush predators (Stuginski *et al.,* 2018). Foraging activity can vary widely among active hunting snake species, depending on the kind of prey hunted (Stuginski *et al.*, 2018), we chose to reflect a specialization on larger prey as it may be favoured over small prey items (*e.g*., Purwandana *et al.,* 2016; Wallace & Leslie, 2008). As such, this will lead to longer use of sheltering sites (for thermoregulation and digestion of prey items).

| Data stream | State 1 - Sheltering | State 2 - Moving | State 3 - Resting |
| --- | --- | --- | --- |
| Step length | *s_1_ ∼ gamma* (0.01, 0.01) | *s_2_ ∼ gamma* (20, 10) | *s_3_ ∼ gamma* (0.2, 0.2) |
| Turning angle | *s_1_ ∼ von Mises* (0, 0.01) | *s_2_ ∼ von Mises* (0, 0.01) | *s_3_ ∼ von Mises* (0, 0.01) |

The transition probability matrix for Species 1 is the following:

Γ =$\left( \begin{matrix} -5 & -100 & -10 & 0 & -100 & 0 \\ 6 & 0 & -4 & 0 & 0 & 0 \\ -6 & 0 & 5 & 0 & 0 & 0 \\ 0 & 0 & 0 & 4 & 0 & -4 \\ 0 & 0 & 0 & 2 & 0 & -2 \end{matrix} \right)$

We set the transition probabilities between states 1→2 and 2→1 as a *cosinor* function fluctuating every 21 days ($\tau_{i}$), simulating a pattern of long-term sheltering (~2-3 weeks).

## Species 2

## “*Ambush predator, long-distance moves, long-term sheltering*”

Sedentary, “sit-and-wait” ambush predators include many snake species, such as vipers, pythons, boas, as well as some colubrids and elapids (Shine, 1980; Stuginski *et al.,* 2018). Snakes with an ambush foraging strategy consume a wide range of meal sizes (Glaudas *et al.,* 2019): We chose to model Species 2 after a specialization on larger prey and thus longer digestion periods. The main component of an ambush predator is spending long periods in the same location (Reinert *et al.,* 1984; Webb & Shine, 1997). “Sit-and-wait” predators also usually feed less frequently than active hunters (Huey & Pianka, 1981).

| Data stream | Sheltering | Moving | Resting |
| --- | --- | --- | --- |
| Step length | *s_1_ ∼ gamma* (0.01, 0.01) | *s_2_ ∼ gamma* (15, 5) | *s_3_ ∼ gamma* (0.2, 0.2) |
| Turning angle | *s_1_ ∼ von Mises* (0, 0.01) | *s_2_ ∼ von Mises* (0, 0.01) | *s_3_ ∼ von Mises* (0, 0.01) |

The transition probability matrix for Species 2 is the following:

Γ =$\left( \begin{matrix} -13 & -100 & -11 & 0 & -100 & 0 \\ 5.5 & 0 & -4 & 0 & 0 & 0 \\ -5.5 & 0 & 5 & 0 & 0 & 0 \\ 0 & 0 & 0 & 4 & 0 & -4 \\ 0 & 0 & 0 & 2 & 0 & -2 \end{matrix} \right)$

We set the transition probabilities between states 1→2 and 2→1 as a *cosinor* function fluctuating every 21 days ($\tau_{i}$), simulating a pattern of long-term sheltering (~2-3 weeks).

## Species 3

## “*Ambush predator, short-distance moves, short-term sheltering*”

In contrast to Species 2, we chose to model Species 3 with a smaller body mass, reflected by short-distance moves and specialization on smaller prey (resulting in shorter sheltering events, as digestion is faster with smaller prey items). In particular, species 3 behaves similarly to certain pit vipers (family Viperidae): ambush predators, with low energetic requirements, infrequent feeding events, as well as a reduced movement rate and home range size compared to larger snake species (Mushinsky, 1987; Macartney *et al.,* 1988).

| Data stream | Sheltering | Moving | Resting |
| --- | --- | --- | --- |
| Step length | *s*_1_ ∼ Gamma(0.01, 0.01) | *s*_2_ ∼ Gamma(2, 5) | *s*_3_ ∼ Gamma(0.5, 0.5) |
| Turning angle | *s*_1_ ∼ von Mises(0, 0.01) | *s*_2_ ∼ von Mises(0, 0.01) | *s*_3_ ∼ von Mises(0, 0.01) |

The transition probability matrix for Species 3 is the following:

Γ =$\left( \begin{matrix} -5 & -100 & -5 & 0 & -100 & 0 \\ 2 & 0 & -2 & 0 & 0 & 0 \\ -2 & 0 & 2 & 0 & 0 & 0 \\ 0 & 0 & 0 & 4 & 0 & -4 \\ 0 & 0 & 0 & 2 & 0 & -2 \end{matrix} \right)$

We set the transition probabilities between states 1→2 and 2→1 as a *cosinor* function fluctuating every 7 days ($\tau_{i}$), simulating a pattern of short-term sheltering (~1 week), in opposition of the previous two species (Siers *et al.*, 2018).

# Additional file 1.2

In this paper, we were interested in evaluating how regime and method affected the F-measures of the three archetype reptile species. We ran Bayesian mixed effects models using the *brms* R package (Bürkner, 2017), as to estimate the expected values and the uncertainty associated with each parameter. We built three model sets (one for each species), and each model ran with six independent Markov chains, of 6000 iterations each, discarding the first 1000 iterations per chain as burn-in.

In order to account for the individual variation, we included individual (IND) as a random effect. The variation of F-measures between different methods may not identical for each individual; to address this issue, we added a varying slope for the effect of method (1 + METHOD | IND). To eliminate the number of divergent transitions that could cause bias in the posterior samples, we increased the adapt delta from 0.8 (default) to 0.99.

We tested three different models for each species: 1) Model 1, *null model*, with intercept only; 2) Model 2, with method and regime as group effects; and 3) Model 3, with method and regime as group effects and an added interaction effect.

For model selection, we used the leave-one-outcross-validation with Pareto smoothed importance sampling (PSIS-LOO CV; Vehtari *et al*., 2016) This method is implemented with the function loo(), calculating the expected log pointwise predictive density (ELPD), providing an estimate of a model’s predictive accuracy. It is a better evaluation method than simpler estimates like AIC (Akaike information criterion) and DIC (deviance information criterion). The diagnostic used to determine whether a point is influential is the pareto-k ($\hat{k}$) parameter, with any $\hat{k}$ value above 0.7 considered problematic. To address this issue, we refitted all models by retaining each influential observation at a time (*i.e.,* loo() function with reloo = TRUE).

We then conducted model validation by posterior predictive checks using function pp_check(), to show how close our simulated samples were to the observed data. Finally, we calculated the effect size (Bayesian R^2^; Gelman *et al.*, 2018).

## Species 1

## “*Active hunter, long-distance moves, long-term sheltering*”

An initial run for species 1 revealed model 2 with one observation with a $\hat{k}$ > 0.7, and model 3 with two such observations. After re-running the leave-one-outcross-validation, all $\hat{k}$ estimates are < 0.7; these are the final model comparisons:

| Model | Formula | LOOIC | SE | ΔELPD | ΔSE |
| --- | --- | --- | --- | --- | --- |
| brm3 | METHOD * REG | -3262.4 | 104.0 | 0 | 0 |
| brm2 | METHOD + REG | -2024.3 | 67.9 | -619.1 | 37.6 |
| brm1 | *Null model* | -1643.9 | 71.0 | -809.3 | 36.9 |

Model 3 (METHOD * REG) had the lowest LOOIC values, indicating the highest predictive accuracy. In addition, all parameters have $\hat{R}$ = 1.00 with reasonably large effective sample sizes, strongly suggesting that the sampling algorithm has converged to the posterior distribution. The distribution of the observed values versus simulated values also suggests that the fitted model is doing reasonably well (Figure S1). The visual inspection of the traceplots also confirmed chain convergence. The effect size of model 3 (Bayesian R^2^) was 0.960 ± 0.001 (CrI: 0.958–0.962).


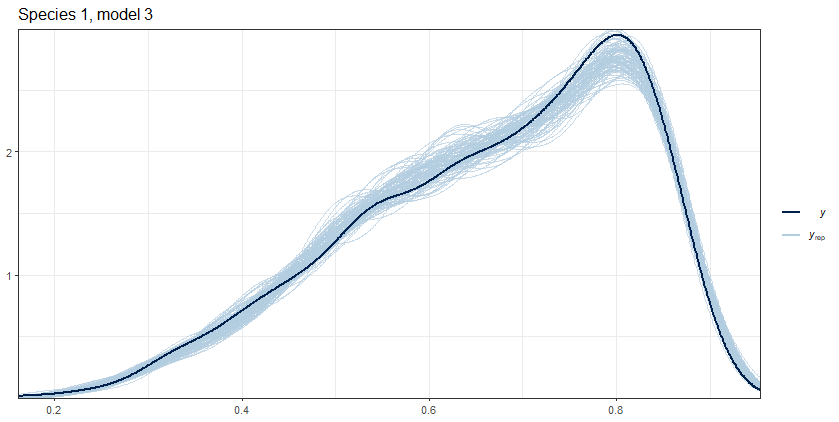


**Figure S1.** Distribution of observed values, compared with distributions of simulated values drawn from the posterior predictive distribution, using Species 1 Model 3, and only showing the first 100 samples for ease of visualization.

## Species 2

## “*Ambush predator, long-distance moves, long-term sheltering*”

An initial run for species 2 revealed model 3 with five observations with a $\hat{k}$ > 0.7. After re-running the leave-one-outcross-validation, all $\hat{k}$ estimates are < 0.7; these are the final model comparisons:

| Model | Formula | LOOIC | SE | ΔELPD | ΔSE |
| --- | --- | --- | --- | --- | --- |
| brm3 | METHOD * REG | -3064.7 | 117.4 | 0 | 0 |
| brm2 | METHOD + REG | -2047.0 | 73.8 | -510.8 | 48.3 |
| brm1 | *Null model* | -1765.3 | 80.9 | -649.7 | 47.9 |

Model 3 (METHOD * REG) had the lowest LOOIC values, indicating the highest predictive accuracy. In addition, all parameters have $\hat{R}$ = 1.00 with reasonably large effective sample sizes, strongly suggesting that the sampling algorithm has converged to the posterior distribution. The distribution of the observed values versus simulated values also suggests that the fitted model is doing reasonably well (Figure S2). The visual inspection of the traceplots further confirmed chain convergence. The effect size of model 3 (Bayesian R^2^) was 0.946 ± 0.002 (CrI: 0.942–0.949).


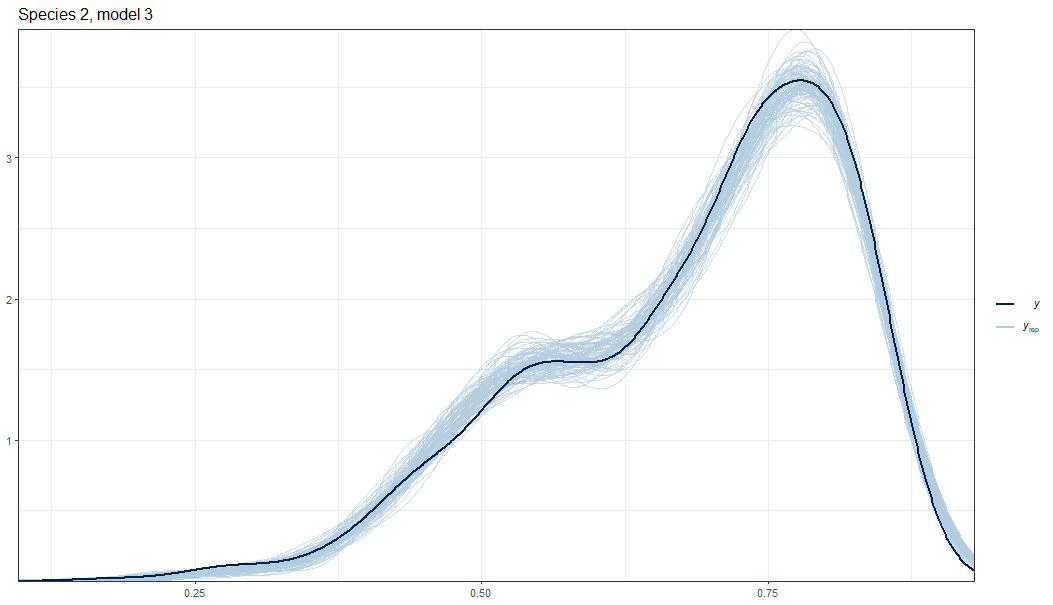


**Figure S2.** Distribution of observed values, compared with distributions of simulated values drawn from the posterior predictive distribution, using Species 2 Model 3, and only showing the first 100 samples for ease of visualization.

## Species 3

## “*Ambush predator, short-distance moves, short-term sheltering*”

An initial run for species 3 revealed model 1 with one observation with a $\hat{k}$ > 0.7, and model 3 with 9 such observations. After re-running the leave-one-outcross-validation, all $\hat{k}$ estimates are < 0.7; these are the final model comparisons:

| Model | Formula | LOOIC | SE | ΔELPD | ΔSE |
| --- | --- | --- | --- | --- | --- |
| brm3 | METHOD * REG | -2956.0 | 81.1 | 0.0 | 0.0 |
| brm2 | METHOD + REG | -2274.5 | 57.3 | -340.8 | 36.3 |
| brm1 | *Null model* | -1967.6 | 66.5 | -494.1 | 40.5 |

Model 3 (METHOD * REG) had the lowest LOOIC values, indicating the highest predictive accuracy. In addition, all parameters have $\hat{R}$ = 1.00 with reasonably large effective sample sizes, strongly suggesting that the sampling algorithm has converged to the posterior distribution. The distribution of the observed values versus simulated values also suggests that thefitted model is doing reasonably well (Figure S3). The visual inspection of the traceplots also confirmed chain convergence. The effect size of model 3 (Bayesian R^2^) was 0.905 ± 0.004 (CrI: 0.897–0.911).

**
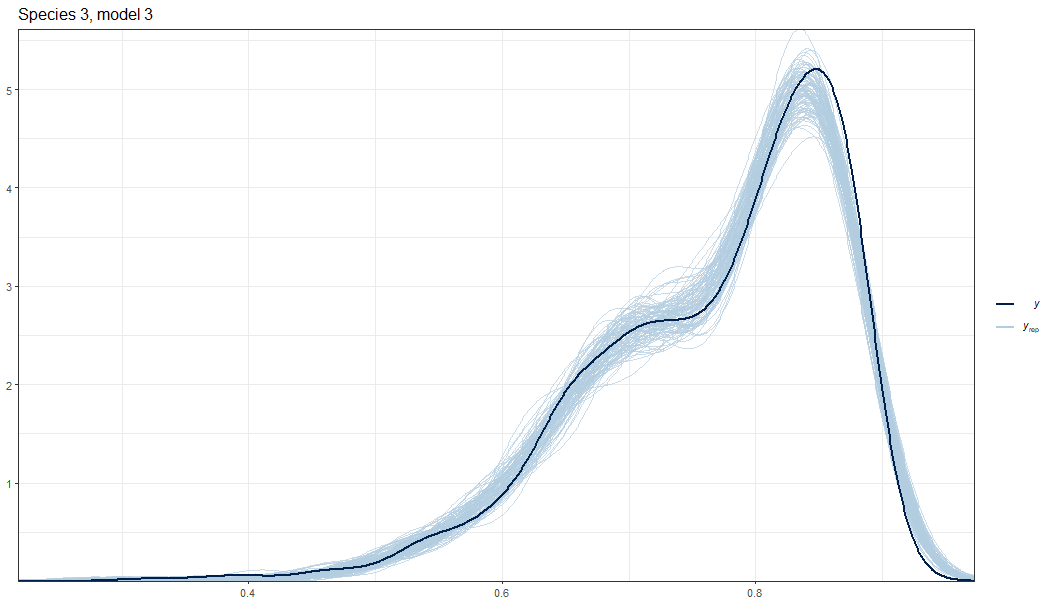
**

**Figure S3.** Distribution of observed values, compared with distributions of simulated values drawn from the posterior predictive distribution, using Species 3 Model 3, and only showing the first 100 samples for ease of visualization.

# Additional file 1.3


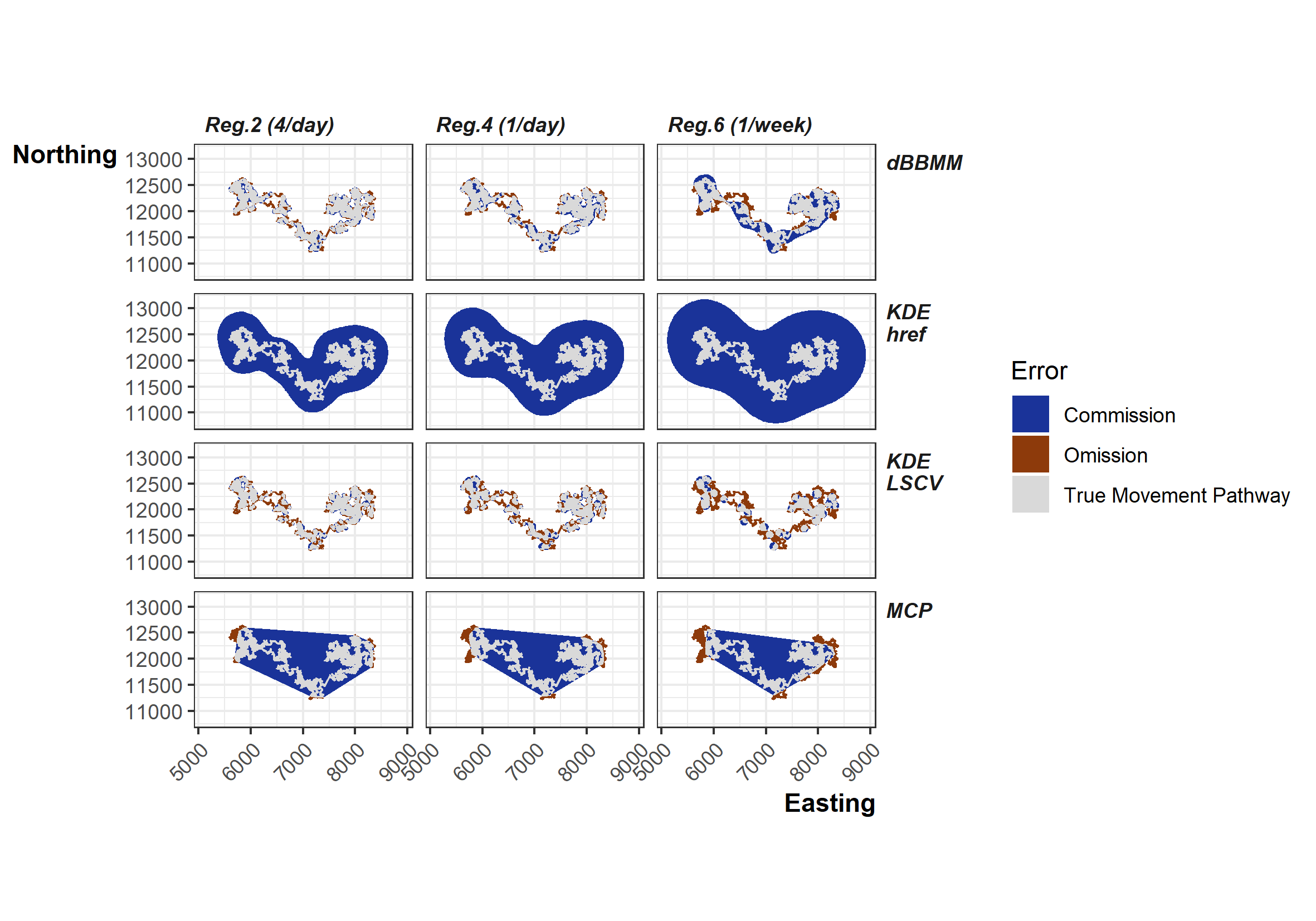


**Figure S4.** An example individual from species 2 of how method and regime can interact to produce different levels of commission (Type I, false negative; blue areas), and omission (Type II, false positive; red areas) error compared to the true movement pathway (grey areas). All contours shown are produced from the 95% occurrence distribution.


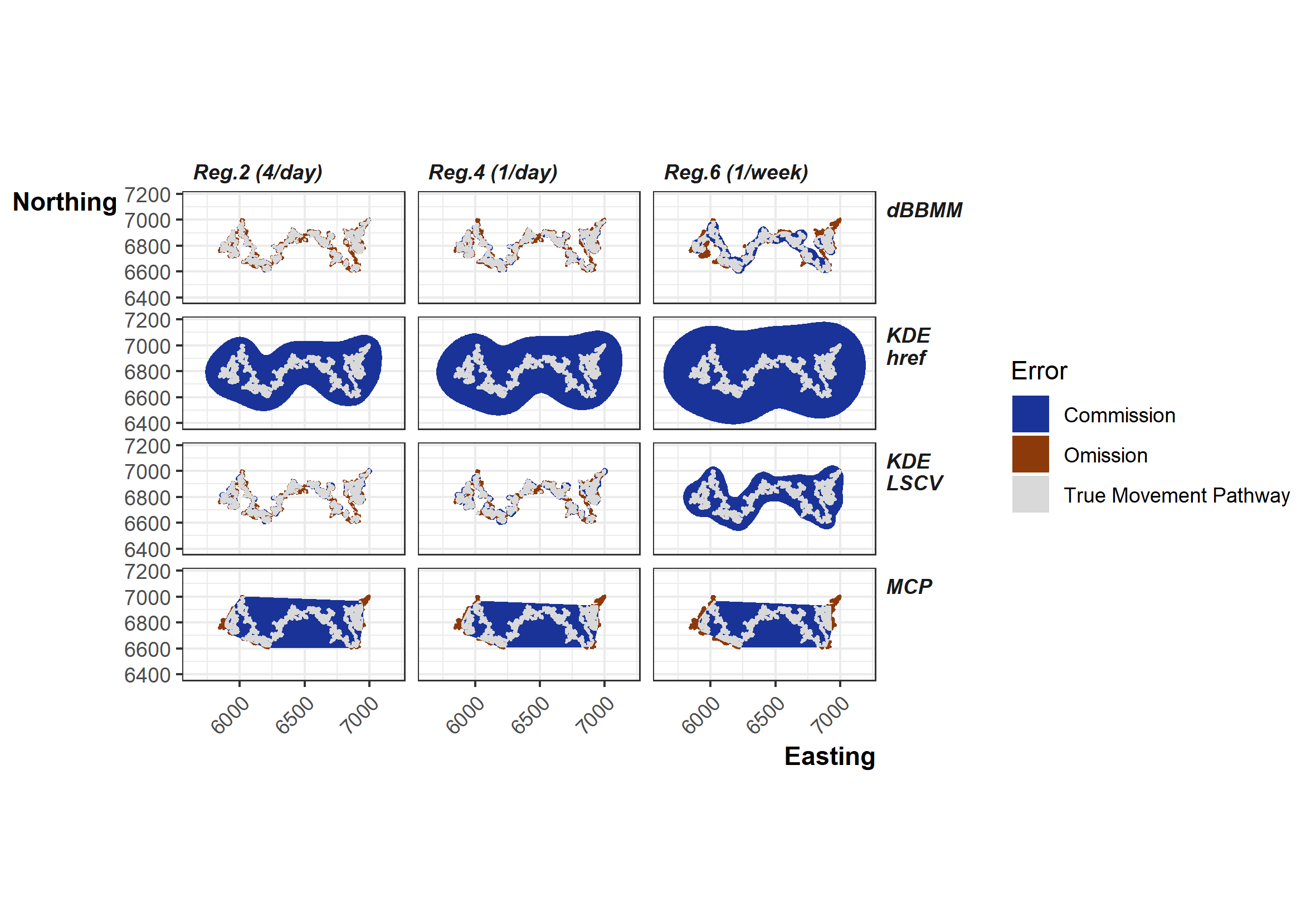


**Figure S5**. An example individual from species 3 of how method and regime can interact to produce different levels of commission (Type I, false negative; blue areas), and omission (Type II, false positive; red areas) error compared to the true movement pathway (grey areas). All contours shown are produced from the 95% occurrence distribution.

# Additional references

Bürkner, P. C. (2017). brms: An R package for Bayesian multilevel models using Stan. Journal of Statistical Software, 80(1), 1-28.

Gelman, A., Goodrich, B., Gabry, J., & Vehtari, A. (2019). R-squared for Bayesian regression models. The American Statistician, 73(3), 307-309.

Glaudas, X., Glennon, K. L., Martins, M., Luiselli, L., Fearn, S., Trembath, D. F., Jelić, D., & Alexander, G. J. (2019). Foraging Mode, Relative Prey Size and Diet Breadth: A Phylogenetically‐Explicit Analysis of Snake Feeding Ecology. Journal of Animal Ecology.

Huey, R. B., & Pianka, E. R. (1981). Ecological consequences of foraging mode. Ecology, 62: 991-999.

King, M. B., & Duvall, D. (1990). Prairie rattlesnake seasonal migrations: episodes of movement, vernal foraging and sex differences. Animal Behaviour, 39(5), 924-935.

Macartney, J. M., Gregory, P. T., & Larsen, K. W. (1988). A tabular survey of data on movements and home ranges of snakes. Journal of Herpetology, 61-73.

Madsen, T., & Shine, R. (1993). Temporal variability in sexual selection acting on reproductive tactics and body size in male snakes. The American Naturalist, 141(1), 167-171.

McClintock, B. T., & Michelot, T. (2018). momentuHMM: R package for generalized hidden Markov models of animal movement. Methods in Ecology and Evolution, 9(6), 1518-1530.

Mushinsky, H. R., Hebrard, J. J., & Vodopich, D. S. (1982). Ontogeny of water snake foraging ecology. Ecology, 63(6), 1624-1629.

Reinert, H. K., Cundall, D., & Bushar, L. M. (1984). Foraging behavior of the timber rattlesnake, Crotalus horridus. Copeia, 976-981.

Secor, S. M., & Nagy, K. A. (1994). Bioenergetic correlates of foraging mode for the snakes *Crotalus cerastes* and *Masticophis flagellum*. Ecology, 75(6), 1600-1614.

Shine, R. (1980). Ecology of the Australian death adder *Acanthophis antarcticus* (Elapidae): evidence for convergence with the Viperidae. Herpetologica, 281-289.

Siers, S. R., Yackel Adams, A. A., & Reed, R. N. (2018). Behavioral differences following ingestion of large meals and consequences for management of a harmful invasive snake: A field experiment. Ecology and Evolution, 8(20), 10075–10093.

Stuginski, D. R., Navas, C. A., de Barros, F. C., Camacho, A., Bicudo, J. E. P. W., Grego, K. F., & De Carvalho, J. E. (2018). Phylogenetic analysis of standard metabolic rate of snakes: a new proposal for the understanding of interspecific variation in feeding behavior. Journal of Comparative Physiology B, 188(2), 315-323.

Vehtari, A., Gelman, A., & Gabry, J. (2017). Practical Bayesian model evaluation using leave-one-out cross-validation and WAIC. Statistics and computing, 27(5), 1413-1432.

Wallace, K. M., & Leslie, A. J. (2008). Diet of the Nile Crocodile (*Crocodylus niloticus*) in the Okavango Delta, Botswana. Journal of Herpetology, 42(2), 361–368.

Webb, J. K., & Shine, R. (1997). A field study of spatial ecology and movements of a threatened snake species, *Hoplocephalus bungaroides*. Biological conservation, 82(2), 203-217.

Webb, J. K., & Shine, R. (1998). Thermoregulation by a nocturnal elapid snake (*Hoplocephalus bungaroides*) in southeastern Australia. Physiological Zoology, 71(6), 680-692.
